# Evaluation of Changes in Protein Quality of High-Pressure Treated Aqueous Aquafaba

**DOI:** 10.3390/molecules26010234

**Published:** 2021-01-05

**Authors:** Fatemah B. Alsalman, Hosahalli S. Ramaswamy

**Affiliations:** 1 Food and Nutrition Program, Environment & Life Sciences Research Center, Kuwait Institute for Scientific Research, P.O. Box 24885, Safat 13109, Kuwait; Fatemah.Alsalman@mail.mcgill.ca; 2 Department of Food Science and Agricultural Chemistry, McGill University, 21,111 Lakeshore Road, Sainte-Anne-de-Bellevue, QC H9X 3V9, Canada

**Keywords:** aquafaba, high pressure processing, protein quantification by ftir, emulsification properties, thermal properties

## Abstract

Chickpea cooking water (CCW), known as aquafaba, has potential as a replacement for egg whites due to its emulsion and foaming properties which come from the proteins and starch that leach out from chickpeas into the cooking water. High pressure (HP) processing has the ability to modify the functional characteristics of proteins. It is hypothesized that HP processing could favorably affect the functional properties of CCW proteins by influencing their structure. The objective of this study to evaluate the effect of HP treatment on the associated secondary structure, emulsion properties and thermal characteristics of CCW proteins. A central composite rotatable design is used with pressure level (227–573 MPa) and treatment time (6–24 min) as HP variables, and concentration of freeze dried CCW aquafaba powder (11–29%) as product variable, and compared to untreated CCW powder. HP improves aquafaba emulsion properties compared to control sample. HP reduces protein aggregates by 33.3%, while β-sheets decreases by 4.2–87.6% in which both correlated to increasing protein digestibility. α-helices drops by 50%. It affects the intensity of some HP treated samples, but not the trend of bands in most of them. HP treatment decreases T_d_ and enthalpy because of increasing the degree of denaturation.

## 1. Introduction

Legumes are good sources of protein, complex carbohydrates and dietary fibre. They contain 17–40% of protein which is equal or superior to the 18–25% protein content in meats [1]. Proteins in pulses are composed mainly of globulins which are soluble in salt solutions and albumins that are soluble in water. Albumins represent only 10–20% of the total proteins in seeds. Although they are characterized by low molecular weights (5–80 kDa), they are the most nutritive proteins in pulse seeds in terms of amino acid composition [2]. Among legumes, chickpea is one of the most important grain legumes in the world [3,4], in addition, its protein and carbohydrate quality are better than those of other pulses [5]. Chickpeas contain 20–26% protein and 43–46% starch [6]. The protein fractions in chickpeas are mainly globulins (56%), glutelins (18%), albumins (12%), and prolamin (3%) [3].

Cooking is the common way to process legumes and the traditional way in food preparation in general. Heat treatment affects protein structures which in turns change their functionality. Functional properties are the chemical and physical properties that change the performance of macromolecules in food systems which in turn reflects the usage and application of those molecules in food industry [2]. Thermal treatment causes protein denaturation due to secondary structure changes [7].

CCW known as aquafaba, has emerged recently as a replacement for egg whites in many food recipes like sponge cakes, meringues and mayonnaises due to its emulsion and foaming properties that comes from the proteins (albumin) and starch which leach out from chickpeas into the water during cooking [8,9,10]. The cooking water of multiple legumes such as white beans, yellow peas, green lentil and chickpeas has good functional properties, especially good foaming capacity, and CCW had the best gelling capacity [11]. Since vegans are a growing community day by day, more of those plant-based replacements are being exploited to provide popular alternatives with some functional enhancements and health benefits.

Novel technologies such as HP processing have shown the ability to modify protein structures which in turn can improve their textural and functional properties [12,13,14]. Majority of such studies have been performed previously evaluating HP processing on uncooked legumes. Not much information can be found in literature on processing cooked legumes. One single study carried out on CCW to evaluate the effects of ultrasound treatment [15], which is also considered as a novel technology, on emulsion and foaming properties. Results indicated enhancement of these properties significantly for varying degrees with positive influence. Therefore, it is hypothesized that HP treatment would favorably affect CCW functional properties by changing protein secondary structure as well as modifying the starch structure and type. Unfortunately, there is no detailed published study on CCW protein/starch structural and functional properties after HP treatment. Because aquafaba is obtained by cooking process, studying differential scanning calorimetry (DSC) is important to see the changes in protein denaturation. Emulsion capacity and stability represented emulsion properties.

The objective of this study was to evaluate the effects of HP processing (227–573 MPa for 6–24 min) on protein secondary structure, emulsion properties and thermal properties of CCW through response surface experimental design and compare them to untreated CCW. Freeze dried CCW was prepared under optimized cooking conditions as suggested from earlier studies [16].

## 2. Results and Discussion

The split-plot central composite RSM design illustrated in Table 1 and Table 2 was employed and fitted four models for all responses varied between quadratic, cubic, linear, and 2FI models which are summarized in Table 3. Details were reduced in some cases to remove the insignificant factors and improve the model. Variables were divided by the software into whole-plot and sub-plot categories. Whole-plot contained the restricted factor, hard to change variable, in our case the pressure level, while sub-plot contains easy to change factors which are in our case pressurization time and aquafaba concentration. Optimization step was not performed since the objective of the study was not to reach a specific maximization or minimization, but to use the model as a screening test to investigate the effect of high pressure treatment and aquafaba concentration on emulsion properties, starch types and secondary structure.

### 2.1. Effect of Variables on Emulsion Properties

Aquafaba can be considered as a Pickering emulsifier which is an emulsion that can be stabilized by solid particles such as starch and protein. Pickering emulsifiers have small particle size and high hydrophobicity in order to reduce the surface tension between the two immiscible liquids rather than surfactants [17]. Some studies have discussed in details the characteristics of Pickering emulsifiers and listed rice, quinoa and amaranth as Pickering emulsifiers while classifying quinoa as the best stabilizer among them since it has higher protein percentage, while amaranth had the highest emulsion capacity [18,19]. Other studies found different food-grade solid particles that can be used as emulsifiers such as oat starch [20], soy protein, whey protein and zein [17]. As a result, aquafaba has Pickering emulsifier characteristics because it consists of a high percentage of starch (24–31%) and proteins (1% wet basis) in addition to possessing high hydrophobicity and small particle size that does not exceed 4 micrometres [11].

In this study, emulsion capacity had a reduced cubic model fitted to the experimental data where the pressure level was insignificant (*p* ≥ 0.05), but the sub-plot was significant (*p* < 0.05) for the concentration variable and the interaction between pressure level and concentration. The variation for that response was not large since it ranged from 4.6 to 5.3 mL. The highest emulsion capacity was for 300 MPa sample which contained 25% aquafaba concentration. The concentration had a positive coefficient shown in Table 4, as a result it affected emulsion capacity positively. No literature was found that treated aquafaba with high pressure, but one study applied ultrasound on aquafaba and concluded that it enhanced emulsion capacity since ultrasound can increase protein partial denaturation which in turn ease the water-oil interface adsorption [15].

**Figure 1:**
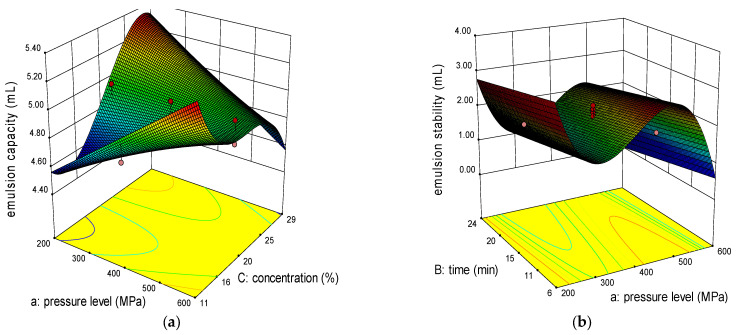

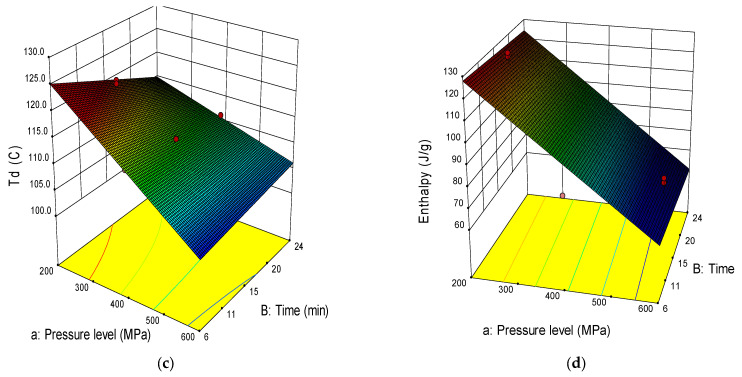
3-D graphs corresponding to models fitted for emulsion capacity (**a**), emulsion stability (**b**), temperature of denaturation (**c**), and enthalpy (**d**).

On the other hand, emulsion stability had a reduced cubic model as illustrated in Figure 1b. Pressure level and pressurization time variables had a significant effect where the stability decreased with longer pressurization time. Stability could be improved with higher pressure levels since time had a negative coefficient and pressure level had positive one illustrated in Table 4. There were no studies conducted on applying HP treatment on cooked ingredients such as aquafaba but applied it either on emulsions then studied their properties or on native starches or proteins. A study on whey protein emulsions applied 100 and 200 MPa and found that higher pressure could decrease the particle size and reduced emulsion creaming and coalescence which in turn increased the stability [21]. By comparing untreated sample, the results from this study asserts the previous study’s findings because there is a significant enhancement in emulsion stability since it has around quarter of HP treated samples. The same study also found that increasing protein concentration had a positive effect on emulsion stability which contradicts the findings in this present study since the concentration did not have a significant effect on emulsion stability, but another study applied high pressure on corn starch that enhanced emulsion properties [22]. Heated pea proteins had better emulsifying properties than unheated proteins was the conclusion of a study which investigated the effect of heat treatment on pea protein emulsifying properties [23].

The reason of our results not being in agreement with some of the other studies might be due to differences in the status of the material. In this study, the aquafaba sample used was a cooked material which is already fully denatured since it was pressure cooked for 60 min, so high pressure would not be expected to demonstrate any significant further denaturation-related effect on the molecules as in other studies which applied HP on raw materials and then studied its effects.

### 2.2. Effect of Variables on Secondary Structure

Protein secondary structure is mainly detectable by the amide I region of the infrared spectrum because it is the most useful part and frequently used for conformational changes such as protein folding/unfolding in addition to the formation of aggregates [24]. Amide I region is 1600–1700 cm^−1^ which is related to C=O stretching of peptide backbone and N-H bending vibrations [25]. It consists of overlapping bands which are α-helix, β-sheets and turns, random coil, and aggregates [26]. Deconvoluting those bands allows to isolate each band and distinguish its position/frequency and intensity to be able to assign it to the right secondary structure component and quantify it [27,28,29,30,31,32]. The assignments of amide I band in the protein are protein aggregates at 1610–1615 cm^−1^, antiparallel β-sheets 1618–1623 cm^−1^, β-strands 1629–1633 cm^−1^, β-sheets 1630–1638 cm^−1^, random coil 1643–1645 cm^−1^, α-helices 1650–1660 cm^−1^, β-turns 1660–1680 cm^−1^, Antiparallel β-sheets 1680–1688 cm^−1^, and β-aggregates 1690–1695 cm^−1^ [2].

#### 2.2.1. Protein Aggregates

Protein aggregates can be found in two regions at the beginning of the amide I region (1610–1615 cm^−1^) called A_1_ and at the end of it (1690–1695 cm^−1^) called A_2_ [2]. A_1_ are inter-molecular aggregates, while A_2_ are intra-molecular aggregates which are related to β-sheets structure [28]. Our findings showed that A_1_ is higher than A_2_ where the former ranged (5.2–9.3%) while the later ranged (0.0–5.7%) for HP-treated aquafaba (Table 2). Control sample had 14.0% and 0.74% for A_1_ and A_2,_ respectively. Pressurization time affected both responses significantly. On the other hand, pressure level was significant for A_1_ results and not for A_2_ results, but its interaction with aquafaba concentration (0.004 *p*-value) in A_1_ and with pressurization time (<0.0001 *p*-value) in A_2_ gave significant results on the responses. Increasing pressure level decreased aggregates from 8% to 5.4% (A_1_) and from 0.8% to 0.0% (A_2_) at 227 MPa and 573 MPa, respectively due to rupturing non-covalent bonds between proteins such as H-bonds or rearranging them.

A study found that raw chickpea has 10.5% and 5.8% of protein aggregates in A_1_ and A_2_, respectively [24]. They also found that after thermal treatment at 120 °C for 30 min A_1_ decreased slightly to 10.3% while A_2_ increased to 6.0%. The control sample in our study which is only treated with heat agrees with the previous statement since it contained the highest protein aggregates percentage compared to HP-treated samples. It has been reported that thermal treatment induced aggregates in A_1_ region and the longer the processing the more aggregates formed, but they are sensitive to shear treatment where those aggregates can be disrupted [19]. There was a study where ultrasound treatment was applied on faba bean protein and found that it could increase the aggregates in A_2_ region and decrease A_1_ aggregates. [27]. Aggregates have a negative correlation with digestibility, but considered as very stable structures due to disulphide bonding [24,33].

#### 2.2.2. Beta Structures (β-Sheets, β-Turns, Antiparallel β-Sheets)

**Figure 2:**
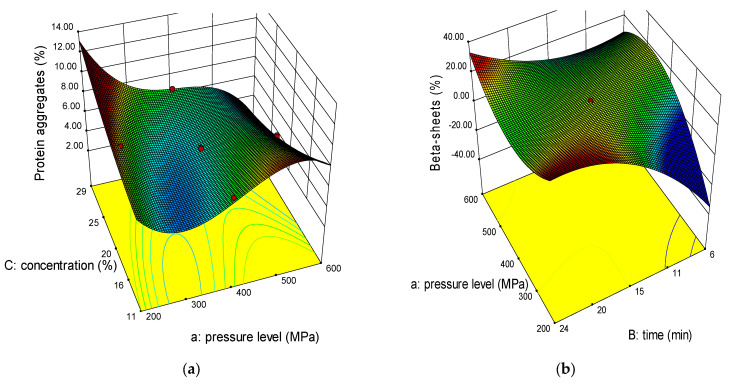

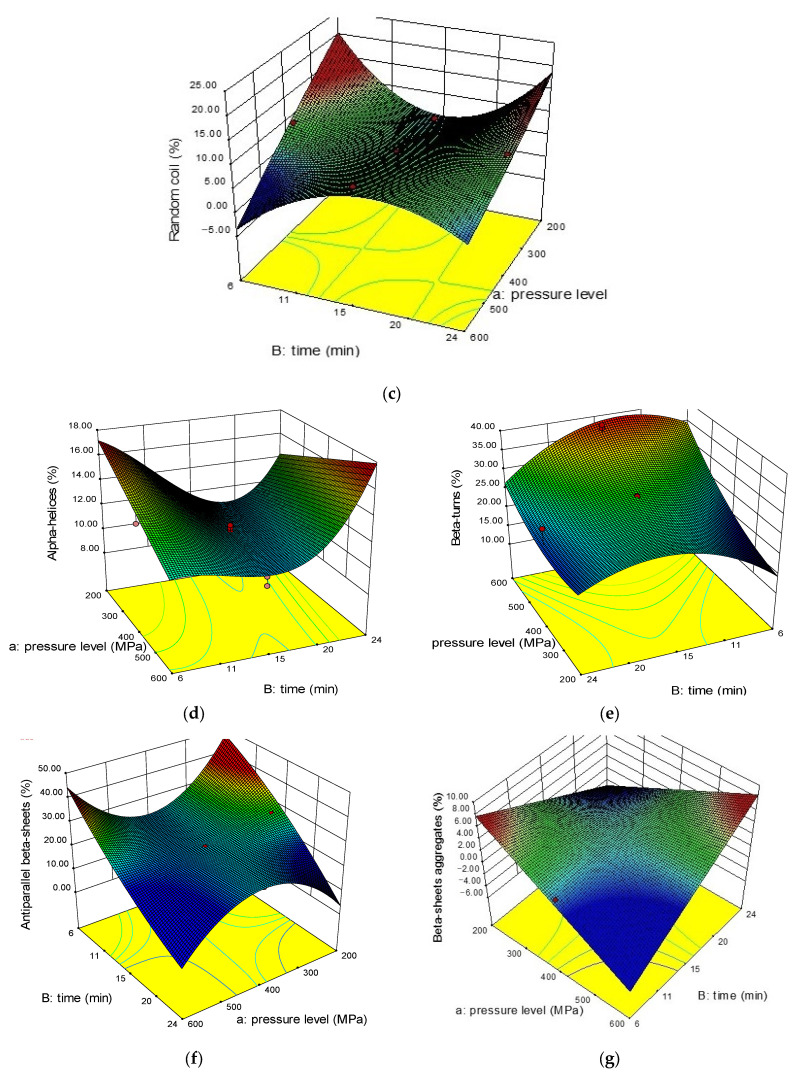
3-D graphs corresponding to models fitted for protein aggregates (**a**), beta-sheets (**b**), random coil (**c**), alpha-helices (**d**), beta-turns (**e**), antiparallel beta-sheets (**f**), and beta-sheets aggregates (**g**) of chickpea water (aquafaba) teated with HP.

Beta (β) structures include many types such as β-sheets, turns, strands and antiparallel β-sheets. Our results showed that β sheets and antiparallel β-sheets have a cubic model, while β-turns has a quadratic model, but all of them with R^2^ ≥ 0.85. From Figure 2b,e,f, it can be noticed that β-turns and antiparallel β-sheets percentages were higher than β-sheets where they ranged 27.7–2.7% (β-sheets), 38.5–18.1% (β-turns), and 39.2–20.0% (antiparallel β-sheets). On the contrary, control sample had higher β-sheets (21.6%) than β-turns (7.99%) and antiparallel β-sheets (6.68%). Among the variables, pressure level was significant to all β-structures, but pressurization time was significant only for β-sheets (0.0110 *p*-value) and antiparallel β-sheets (0.0122 *p*-value). Increasing pressure level decreased the responses since it has a negative coefficient. Regarding aquafaba concentration, it was significant to antiparallel β-sheets only (<0.0001 *p*-value) but its interaction with pressurization level and time was significant for this response and for β-sheets.

It is known that β-structures in most of the legumes, if not all of them, have higher proportions than α-helices which explains the reason of low digestibility in those pulses since beta structures inhibit proteolytic enzymes access [2,30]. A study reported that chickpeas contain 52.1% of β-structures which agrees with our findings where 42.8 was the percentage for β-structures in our control sample and increased to the range 58.3–80.9 for HP treated samples [24]. It has been reported that legumes which contain 7S globulin more than 11S globulin in their protein structure resulted higher β-sheet structure since it was analyzed and proved that 7S globulin contained higher proportion of β-sheets than 11S globulin, which is a reasonable explanation of our findings [31,32].

Further, it has been reported that β-sheets in chickpeas treated at 120 °C for 30 min were 37.7%. On the other hand, β-turns were 19.3% and antiparallel β-sheets were 6.1% which agrees with our control sample for antiparallel β-sheets portion only. That might be because our control sample was thermally treated for 60 min but not 30 min [24]. Ultrasound treatment increased β-structures slightly in faba beans which is similar to our findings where HP treatment increased those structures [27]. A study confirmed that increasing HP treatment to 600 MPa had increased β-turns and shifted the antiparallel β-sheets wavelength [33]. They attributed this change to protein unfolding since β-turns are associated with secondary structure restoration/rebuilding process. Findings from this study agreed with their conclusions where β-sheets decreased from 18.5% to 11.3% and β-turns increased from 24.6% to 38.8% at 227 MPa and 573 MPa, respectively.

#### 2.2.3. Alpha (α) Helices and Random Coils

Random coils and α-helices are usually negatively correlated to each other, where more changes in secondary structure leads to higher random coils and lower α-helices proportions [24]. Table 3 shows that both responses fit a cubic model with R^2^ = 0.99 where the α-helices model was reduced to obtain better results. All variables were significant for both responses except for pressure level in α-helices, but its interaction with aquafaba concentration and pressurization time was significant.

**Figure 3:**
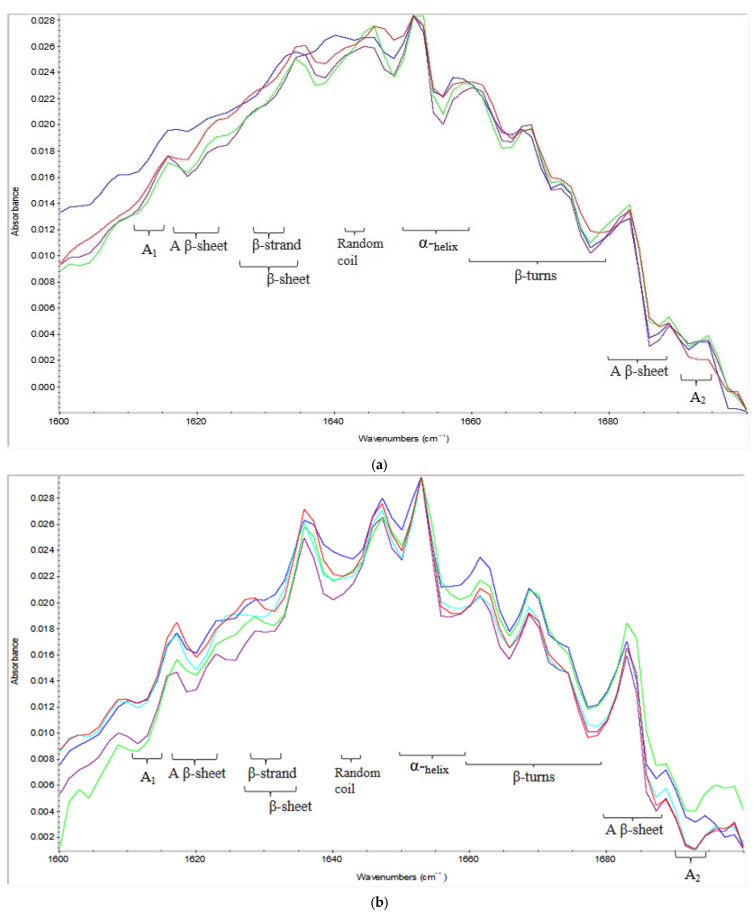

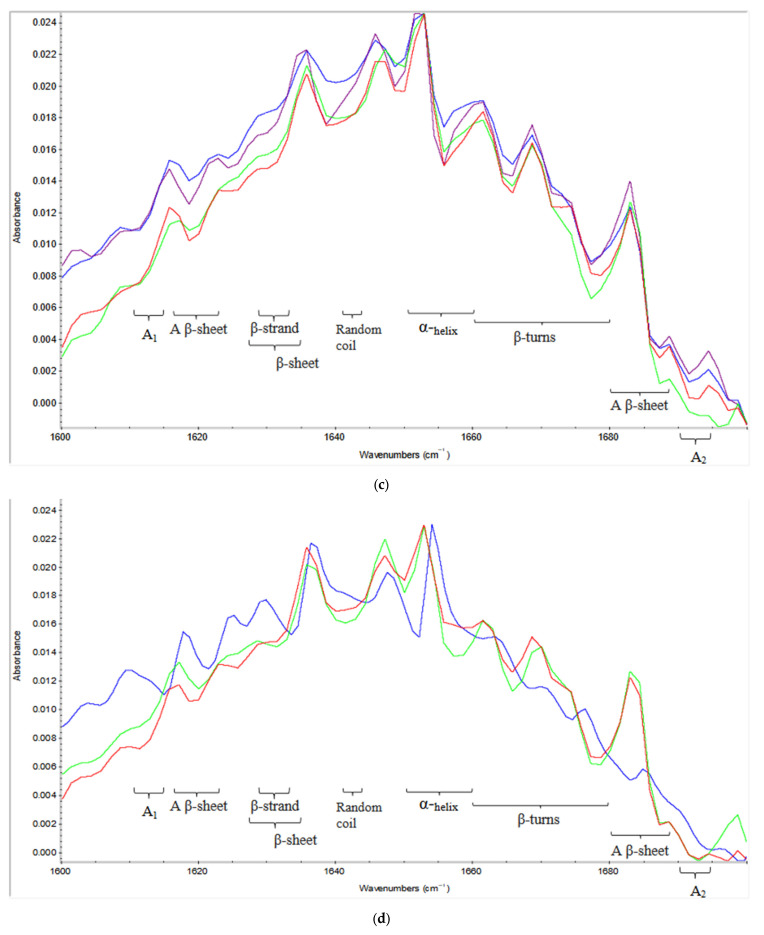
(**a**). FT-IR spectra of HP treated aquafaba samples and control sample. Blue line (Run 1) = 300 MPa for 10 min with 15% aquafaba concentration; Purple line (Run 2) = 300 MPa for 20 min with 25% aquafaba concentration; Green line (Run 3) = 300 MPa for 10 min with 25% aquafaba concentration; Red line (Run 4) = 300 MPa for 20 min with 15% aquafaba concentration; (**b**). FT-IR spectra of HP treated aquafaba samples and control sample. Blue line (Run 19) = 400 MPa for 15 min with 20% aquafaba concentration; Purple line (Run 20) = 400 MPa for 24 min with 20% aquafaba concentration; Green line (Run 21) = 400 MPa for 15 min with 29% aquafaba concentration; Turkuaz line (Run 22) = 400 MPa for 6 min with 20% aquafaba concentration; Red line (Run 23) = 400 MPa for 15 min with 11% aquafaba concentration; (**c**). FT-IR spectra of HP treated aquafaba samples. Blue line (Run 8) = 500 MPa for 20 min with 15% aquafaba concentration; Purple line (Run 9) = 500 MPa for 20 min with 25% aquafaba concentration; Green line (Run 10) = 500 MPa for 10 min with 25% aquafaba concentration; Red line (Run 11) = 500 MPa for 10 min with 15% aquafaba concentration; (**d**). FT-IR spectra of HP treated aquafaba samples and control sample. Blue line = aquafaba without HP treatment (control), red line = HP treated sample at 227 MPa for 15 min with 20% aquafaba concentration, and green line = HP treated sample at 573 MPa for 15 min with 20% aquafaba concentration.

By looking at Table 4, it can be noticed that increasing the pressure level and pressurization time should increase random coils while increasing the concentration decreases the response depending on their coefficients. Examples from Table 2 demonstrate the variability associated with the HP effects. Random coils increased from 5.6% to 8.1% when the pressure level increased from 227 MPa to 573 MPa for the same concentration and pressurization time. In another example, when time was increased from 10 min to 20 min random coils increased from 3.4% to 10.2% at the same pressure level (500 MPa) and concentration (25%). In the last example to show the concentration effect, by increasing the concentration from 15% to 25% random coils decreased from 7.4% to 3.4% at 500 MPa for 10 min. Figure 3b illustrates an obvious decrease in the intensity for samples treated at 400 MPa for 24 min compared to 15 min in the random coil wavenumber section. This is also further proof of increasing random coils with increasing pressurization time.

On the other hand, α-helices decreased significantly after applying HP treatments (Table 2) where the control sample contained 21.2% α-helix structures and decreased to 10.8% and 10.3% at 227 MPa and 573 MPa, respectively. Also, those structures decreased from 16.9% to 11.1% when the concentration was increased from 15% to 25% at 500 MPa for 10 min since the concentration had a negative coefficient. Figure 3c shows the increase in intensity of the α-helices wavenumber section for run #11 (500 MPa, 10 min, 15% concentration) over run #10 (500 MPa, 10 min, 25% concentration) that is considered another proof of the decrease in those structures with increasing the concentration. Pressurization time has also a negative correlation with α-helix structures. When the time was increased from 10 min to 20 min, α-helices decreased from 14.3% to 14.1% at 300 MPa with 25% aquafaba concentration.

The percentage of α-helix structures is known not to exceed 20% in most legumes [24]. The same study found that samples treated at 120 °C for 30 min contained 20.6% α-helices which agrees with our findings since control sample contain 21.2% α-helices. α-Helices of chickpea protein decreased ≈3% when it was heated at 120 °C [29]. Thermally-treated soy glycinins increased random coils 2.2% compared to untreated samples [28]. There was a significant decrease in the α-helices band intensity of HP-treated lentil flour slurry at 350 and 650 MPa compared to control sample which confirms protein unfolding since the α-helices portion decreased [26]. In addition, the decrease in the random coils band intensities of HP-treated samples compared to control was observed, which in turn also agrees to the increase in random coils proportions [26]. HP-treated soy protein isolates showed decreased α-helices protein bands and increased random coils content which is in agreement with our study results [33].

### 2.3. Thermal Properties

Differential scanning calorimetry (DSC) is used to investigate protein denaturation and starch gelatinization of solid and solution samples. Thermogram (DSC diagram) results of high-pressure-treated aquafaba slurry and control sample (unpressurized) are summarized in Table 1 (Supplementary Materials). There was no gelatinization peak for all samples including the control (data not shown) which means that samples were already gelatinized after thermal treatment. Thermal treatment was done before pressure treatment using pressure cooker for 60 min which might have been sufficient to gelatinize all the starch.

The temperature of denaturation (T_d_) was influenced significantly (*p* < 0.05) by pressure level and pressurization time as shown in Table 3. The higher the pressure level and pressurization time was, the lower T_d_ (Table 1) which agrees with multiple studies [29,34,35,36]. The endothermic peak’s range of T_d_ was 120.6 °C (control)—103.5 °C (573 MPa) which might be attributed to either protein denaturation or melting of amylose-lipid complexes that were formed during starch gelatinization [12,35,37].

During thermal treatment of the control sample, the temperature of denaturation (T_d_) increased compared to raw (untreated) samples because of the immense uptake of heat that is illustrated through the endothermic peak in DSC thermograms [38]. The increase in T_d_ at 227 MPa to a degree higher than the control sample was due to the rupture of disulphide bonds of proteins which led to the exposure of hydrophobic sites and therefore aggregation which affords a more compact structure with higher thermal stability [39]. Lower T_d_ in other samples treated with higher pressure levels might be due to partial protein denaturation and interactions between the formed complexes [25,35]. Also, the presence of starch and fat in samples may contribute to a lowering or increasing of T_d_ [30,40]. Aquafaba samples contain albumin and globulin factions [9,41] and was reported to have T_d_ in the range of 83–110 °C [20,30].

Regarding the enthalpy of denaturation (∆H), it is the energy that is required to break down molecules and it decreases with increasing pressure intensity which is an indication of protein denaturation [42]. In our case, ∆H decreased linearly with increasing pressure level (*p* = 0.0014) which agrees with many studies [29,30,34,36,38,43,44], while pressurization time and concentration did not contribute significantly (*p* > 0.05) (Table 3). On the contrary, a study conducted on high pressure-treated chickpea flour slurry reported a significant increase in ∆H when the concentration was increased [34]. ∆H ranged 145.2 J/g (control)—71.8 J/g (573 MPa).

The degree of denaturation (DD) increased linearly with pressure intensity. DD ranged from 99.1% for the sample treated at 227 MPa to 99.5% for the sample treated at 573 MPa. A study conducted on soybean proteins proved that DD ranged from 27.8% (200 MPa) to 80.6% (400 MPa) and 84.3% (600 MPa) [45]. Another study reported that the DD of amaranth proteins was 93% at pressures ≥400 MPa [46]. The decrease in ∆H indicates partial denaturation, as protein turns into an unfolded state, where less heat energy (less ∆H) is required to denature the protein [20,47]. It can be also ascribed to disruption of hydrogen bonds, breaking hydrophobic interactions, and protein aggregation [30,47]. ∆H represents melting of amylopectin crystallites which reflects double helices bonding forces that form amylopectin crystallites [48], so samples with higher amylopectin crystallites such as waxy rice flour were less sensitive to high pressure (higher ∆H) than corn starch which contains higher amylose content.

### 2.4. Effect of Variables on Protein Bands

**Figure 4:**
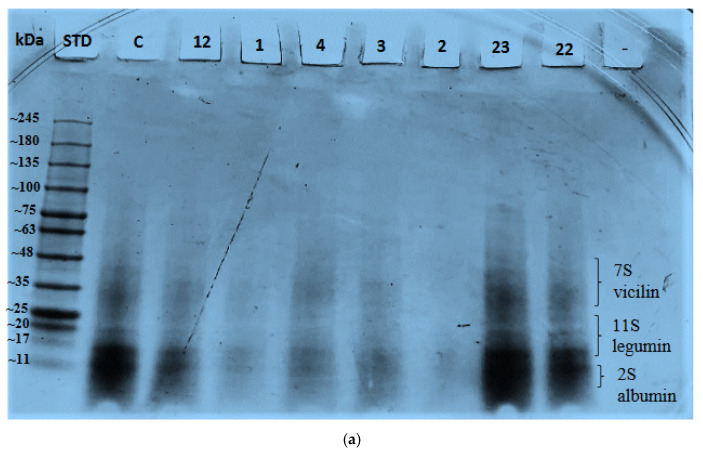

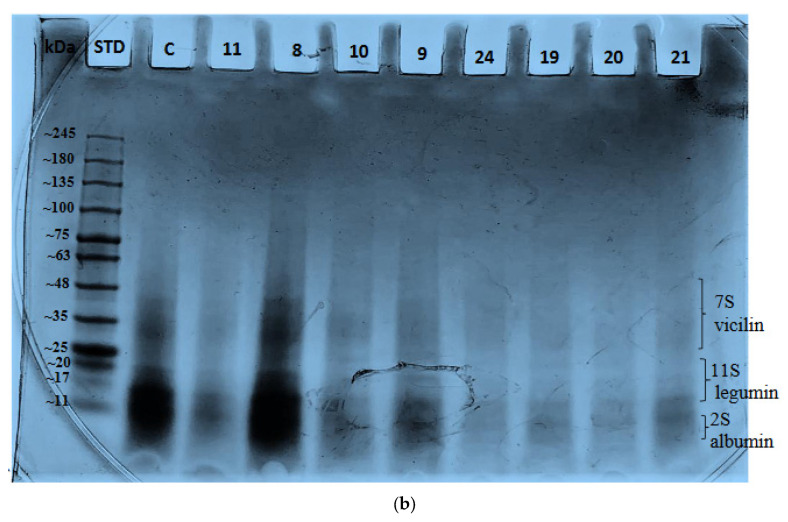
(**a**). SDS-PAGE of HP treated aquafaba proteins. STD = standard proteins; column: C = control (untreated aquafaba); Run 12 = 227 MPa for 15 min with 20% concentration; Run 1 = 300 MPa for 10 min with 15% concentration; Run 4 = 300 MPa for 20 min with 15% concentration; Run 3 = 300 MPa for 10 min with 25% concentration; Run 2 = 300 MPa for 20 min with 25% concentration; Run 23 = 400 MPa for 15 min with 11% concentration; Run 22 = 400 MPa for 6 min with 20% concentration; (**b**). SDS-PAGE of HP treated aquafaba proteins. STD = standard proteins; column: C = control (untreated aquafaba); Run 11 = 500 MPa for 10 min with 15% concentration; Run 8 = 500 MPa for 20 min with 15% concentration; Run 10 = 500 MPa for 10 min with 25% concentration; Run 9 = 500 MPa for 20 min with 25% concentration; Run 24 = 573 MPa for 15 min with 20% concentration; Run 19 = 400 MPa for 15 min with 20% concentration; Run 20 = 400 MPa for 24 min with 20% concentration; Run 21 = 400 MPa for 15 min with 29% concentration.

HP treatment affects protein functionality and digestibility by modifying the protein structure. It also enhances the exposure of polypeptides to digestive enzymes which in turn improves hydrolysis and digestibility [49]. A comparison between untreated aquafaba and HP-treated samples was conducted to see how HP treatment can affect peptide bonds. Figure 4a,b show that detectable bands had molecular weights <48 kDa. There were five visible bands ≈43–45, ≈35, ≈20, ≈15–17, and ≈10–11 kDa. There were significant band intensity differences among the bands due to the different pressure levels, holding time and aquafaba concentration. The densest bands were in lane 23 (Figure 4a) and lane 8 (Figure 4b) in addition to the control sample.

A study that compared raw and boiled chickpeas found that raw seeds contain 46 bands while boiled samples have 35 bands [50]. Since our samples are cooked residue water of chickpeas, aquafaba, the other undetectable bands might be retained in chickpeas. Studies reported that chickpea protein bands are mainly around 22, 23, 24, and 25 kDa which correspond to subunits of 11S legumin and around 33, 34, 37, 40 and 46 kDa which correspond to 7S vicilins with 2S albumins at the lowest molecular weights [51,52].

There were just two studies that were carried out on aquafaba studying peptide bonds by SDS-PAGE where both used canned aquafaba [9,41]. Aquafaba contained seven bands at 10, 12, 15, 23, 39, 51 and 99 kDa which is pretty similar to our study, especially the low molecular weight bands [41]. Those bands were attributed to 2S albumin (10 and 12 kDa), γ-subunit of 7S vicilin (16 kDa), and 11S legumin (23 and 39 kDa). On the other hand it was found that aquafaba contained 11 bands, where most of them are heat-soluble hydrophilic proteins (16.7, 15.7, and 13.2 kDa) and heat-shock proteins (10.1 kDa) [9]. Other bands were oxidoreductase (36.3 kDa), dehydrin (20.4 kDa), and histone (15.4, 14.6, and 14.9 kDa).

In our study we found that bands from the control sample were present in HP-treated aquafaba in the same pattern, which shows the stability of those proteins that might be due to denaturation due to thermal processing [41]. Some bands in lanes 22 and 23 (Figure 4a) and lanes 8, 9, and 21 (Figure 4b) showed higher intensity than other bands. This could be caused by the pressure level at 500 MPa and 400 MPa that caused modifications in covalent bonds such as disulphide bonds and in non-covalent bonds such as denaturation and aggregation of proteins. A high concentration of aquafaba might play a role in band visibility [36]. Other bands that showed lower intensity or even diminished might be due to protein denaturation and/or degradation [36,53].

## 3. Materials and Methods

### 3.1. Materials

Dry Canadian Kabuli chickpeas (CLIC brand) packed in heat sealed clear plastic bags in 407 g portions were purchased from the Provigo Distribution Centre Outlet (Montreal, QC, Canada) and stored at room temperature until used for experiments (a time span of less than a month).

### 3.2. Sample Preparation

Dried chickpeas were soaked at 40 °C for 2 h. then placed in a classic Hawkins brand pressure cooker with 1.5:3.5 chickpea to water ratio and cooked for 60 min. After cooling, it was placed in the freezer (−20 °C) overnight. Then, samples were freeze dried at −30 °C with 13 Pa vacuum at room temperature using pilot scale freeze dryer (SP Scientific/Virtis MR-145BA, Warminster, PA, USA) and stored in sealed containers at 4 °C until further use.

### 3.3. High Pressure (HP) Treatment

HP treatments were given in two different HP units; the first one was a laboratory scale HP system (ACIP 6500/5/12VB-ACB Pressure Systems, Nantes, France) consisting of a cylindrical pressure chamber of (5 L) volume. The pressure-time (P-t) program was designed using a computer connected to a data logger (SA-32, AOIP, Nantes, France). The pressure transmission medium used was water. The compression rate was set at 5 MPa/s up to reaching the desired pressure level for specific holding time followed by a rapid decompression (<4 s) to atmospheric pressure. This equipment could be operated up to 650 MPa, but for this study it was used for pressure levels up to 500 MPa. All treatments were given approximately at room temperature. It was typical that the chamber pressurization would increase the temperature of the pressurization medium approximately 3 °C for every 100 MPa elevation of temperature. With the jacket maintained at room temperature, the actual temperature raise during the maximum pressure was less than 10 °C and when this maximum pressure level used, samples were loaded at 15–20 °C depending on the pressure level to allow equilibration to room temperature. The second HP equipment was a multi-vessel hydrostatic pressure kinetic unit, a High Pressure Multi-Vessel Kinetic unit (U111 apparatus, Unipress, Warsaw, Poland) equipped with a thermal bath. This system could operate at pressures up to 700 MPa and at temperatures varying from 25 to 120 °C. The pressure come up times varied from 40 to 60 s depending on the selected pressure level as higher pressure level required longer come up time (5 MPa/s). The depressurization time was less than 25 s. This HP system was used for pressure level 573 MPa. In the multi-vessel unit the sample chambers were small (5 mL) and made out of metal (beryllium) and the any generated adiabatic heat was quickly dissipated and equilibrated to the bath temperature so the control of temperature was much easier. Freeze dried samples were mixed with water to get a specific percentage according to the experimental design and kept for 1 h at room temperature (25 °C) for hydration prior to HP treatment. HP treatments were given at 5 pressure levels: 227, 300, 400, 500, and 573 MPa, and each pressure, a single pressure cycle (pressure come-up, hold, depressurize) with different holding time between 6 to 24 min depending on the experimental design.

### 3.4. Emulsion Capacity and Stability

Emulsion capacity of aquafaba samples was determined as the procedure used for fish protein isolates with some modifications [54]. Aquafaba samples (1 mL) were diluted 1:8 with water then homogenized for 1 min using tissue tearor (model 985-370, Biospec Products, Inc., Racine, WI, USA). After that (5 mL) of canola oil was added to 5 mL of the homogenized liquid and homogenized again for 2 min using the same homogenizer. Then, the homogenized liquid was centrifuged at 3000 rpm for 30 min and the oil was separated, and emulsion formed was measured by a pipette. Emulsion stability was measured according to the procedure used in faba beans samples’ analysis [55]. The same emulsion previously formed was warmed in a water bath at 80 °C for 30 min, cooled to room temperature, centrifuged at 3000 rpm for 30 min, and the volume of emulsion was measured the same way described previously.

### 3.5. Fourier Transform Infrared (FTIR) Spectroscopy

The FTIR spectra of freeze-dried HP treated aquafaba samples and un treated sample were obtained by using a Manga System 550 FT-IR Spectrometer (Agilent 5500a, Santa Clara, CA, USA) over a wavelength range of 400–4000 cm^−1^ equipped with an OMNIC operating system software (Version 7.3, Thermo Electron Corporation, Waltham, MA, USA). Samples were covered on the surface in contact with attenuated total reflectance (ATR) on a multi-bounce plate of Zn-Se crystal at 25 °C. All spectra were background corrected using an air spectrum, which was renewed after each scan. Each spectrum was collected from an average of 32 scans with a resolution of 4 cm^−1^ and the results were reported as mean values. Fourier self-deconvolution (FSD) was performed and then the peaks were fitted in amide-I region (1700–1600 cm^−1^) since it is the most responsive to the secondary structure of the protein. Gaussian peaks could be assigned to their corresponding structure based on their centre and the integral of each peak was divided by the sum of all determined peaks to identify the proportion (%) of each structure [56].

### 3.6. Thermal Properties

Differential scanning calorimetry (DSC, (TA Q 100, TA Instruments, New Castle, DE, USA) was used to measure the thermal analysis for freeze dried aquafaba samples. The DSC was calibrated with indium for temperature and heat capacity calibration. Aquafaba slurry (6–15 mg) were run from 20 to 200 °C at a 10 °C/min heating ramp in a nitrogen atmosphere (flow rate, 50 mL/min) to detect gelatinization and protein denaturation points. An empty pan was used as a reference. The DSC measurements were done in triplicate. Instrument software (version 4.5A, TA Instruments) was used to calculate thermal properties. Degree of denaturation (%) was calculated as

[100 − (ΔH_pres_/ΔH_atm_)]

where ΔH_pres_ is the enthalpy of pressurized samples and ΔH_atm_ is the enthalpy of samples under atmospheric pressure (unpressurized) [57].

### 3.7. Sodium Dodecyl Sulphate Polyacrylamide Gel Electrophoresis (SDS-PAGE)

Electrophoresis was done by using Mini-PROTEAN II Electrophoresis Cell unit (BIO-RAD, Mississauga, ON, Canada). This unit was connected to electrophoresis power supply (Bio-Rad, Hercules, CA, USA). Freeze dried aquafaba samples were filtered through ultrafiltration centrifuge tubes to purify and concentrate the proteins with molecular weights ≤50 KDa. Then, protein fractions (10 uL) were mixed with (20 uL) of sample buffer containing 62.5 mM Tris-HCl, pH 6.8, 2% SDS, 25% (*v/v*) glycerol, 0.01% bromophenol blue and 5% β-mercaptoethanol. These samples were boiled for 5 min, cooled, and centrifuged for 10 min at 14,000 rpm. 15 uL of supernatant (1.5 ug of protein) was loaded to a sample well of a 4–20% SDS-polyacrylamide gel. The proteins were separated in the gel using 100 V for 1.5 h in running buffer containing 2.5 mM tris, 19.2 mM glycine and 0.01% SDS. The gels were then stained in standard commassie blue-methanol-acetic acid solution for 30 min at RT. Gels were then washed with destaining solution (40% methanol, 10% acetic acid and 50% water).

### 3.8. Experimental Design

A split-plot central composite RSM design was used to investigate the effect of three factors, (pressure level, pressurisation holding time, and aquafaba powder concentration “%”) on thirteen responses (emulsion capacity, emulsion stability, rapidly digested starch, slowly digested starch, total digestible starch, resistant starch, protein aggregates, random coil, beta-sheets, alpha-helices, beta-turns, beta-sheets aggregates, and antiparallel beta-sheets). Twenty-five combinations of the variables were selected by experimental design as shown in Table 1 and Table 2. Another separate experiment of untreated aquafaba was used as control sample to compare the results.

### 3.9. Statistical Analysis

All data was analyzed using the StatEase Design Expert 10.0.5 statistical software (StatEase Inc., Minneapolis, MN, USA). In the procedures employed, the software was used to analyze the test data obtained through experiments by least square multiple regression analysis. Different models, interactions tested, and their suitability was evaluated based on the analysis of variance (ANOVA) and associated F-values. The significance was tested at 5% probability level. The generated statistical parameters were used to assess the validity of generated models.

## 4. Conclusions

HP improved the aquafaba emulsion capacity and stability compared to control samples. It was proved by DSC through increasing the degree of denaturation that is a reflection of higher hydrophobicity which enhanced the emulsion properties. HP could also reduce protein aggregates by 33.3%, while β-sheets decreased by 4.2–87.6%, which are both correlated to increased protein digestibility. α-Helices dropped by 50%. It also affected the intensity of some HP-treated samples, but bands did not disappear. Being able to enhance protein digestibility will in turn improve protein absorption.

## Figures and Tables

**Table 1:** Split-plot central composite RSM design matrix with un-coded values of the factors, emulsion properties and starch types responses.

Run	A: Pressure Level	B: Time	C: Concentration	Emulsion Capacity	Emulsion Stability	T_d_	Enthalpy
	MPa	Min	%	mL	mL	°C	J/g
1	300	10	15	4.83 ± 0.24	1.80 ± 0.25	118.0 ± 3.0	125.4 ± 2.7
2	300	20	25	5.33 ± 0.12	1.30 ± 0.24	114.9 ± 3.4	125.6 ±0.6
3	300	10	25	5.27 ± 0.21	1.50 ± 0.21	120.5 ± 0.4	119.6 ± 2.9
4	300	20	15	4.73 ± 0.21	0.90 ± 0.12	114.9 ± 2.3	111.9 ± 3.1
5	400	15	20	4.90 ± 0.08	2.00 ± 0.21	112.1 ± 4.6	87.7 ± 1.1
6	400	15	20	5.00 ± 0.07	2.30 ± 0.21	110.5 ± 4.5	87.1 ± 1.3
7	400	15	20	4.80 ± 0.08	1.80 ± 0.20	111.9 ± 4.7	88.0 ± 1.3
8	500	20	15	5.10 ± 0.08	2.43 ± 0.05	114.0 ± 0.7	80.4 ± 4.8
9	500	20	25	5.10 ± 0.08	2.40 ± 0.43	112.8 ± 4.3	83.8 ± 2.2
10	500	10	25	5.00 ± 0.08	2.53 ± 0.21	113.1 ± 2.0	86.4 ± 2.0
11	500	10	15	4.83 ± 0.24	2.67 ± 0.24	115.2 ± 1.1	87.9 ± 0.2
12	227	15	20	4.70 ± 0.29	2.10 ± 0.46	119.9 ± 0.4	127.0 ± 1.8
13	227	15	20	5.00 ± 0.30	2.30 ± 0.45	120.6 ± 0.3	125.2 ± 1.7
14	400	15	20	4.90 ± 0.08	2.30 ± 0.29	110.8 ± 4.0	88.4 ± 0.9
15	400	15	20	4.80 ± 0.07	2.20 ± 0.29	108.2 ± 4.3	87.5 ± 1.1
16	400	15	20	5.00 ± 0.08	2.00 ± 0.30	112.9 ± 4.6	88.9 ± 1.0
17	400	15	20	4.90 ± 0.08	1.60 ± 0.29	109.7 ± 4.4	87.1 ± 1.1
18	400	15	20	4.80 ± 0.09	2.10 ± 0.31	110.3 ± 4.3	89.4 ± 0.9
19	400	15	20	5.00 ± 0.07	2.20 ± 0.28	108.3 ± 4.5	88.3 ± 1.0
20	400	24	20	4.60 ± 0.08	1.40 ± 0.12	111.4 ± 2.4	82.4 ± 3.1
21	400	15	29	4.83 ± 0.12	2.00 ± 0.12	112.0 ± 0.4	91.7 ± 3.4
22	400	06	20	4.93 ± 0.09	1.90 ± 0.43	113.8 ± 1.9	80.6 ± 3.0
23	400	15	11	4.80 ± 0.08	1.10 ± 0.12	112.5 ± 4.8	86.0 ± 0.6
24	573	15	20	4.83 ± 0.24	1.20 ± 0.08	105.0 ± 3.5	71.8 ± 2.6
25	573	15	20	5.00 ± 0.23	1.30 ± 0.09	103.5 ± 3.6	74.1 ± 2.4
Control	--	--	20	5.00 ± 0.06	0.60 ± 0.17	115.7 ± 0.3	145.2 ± 4.2

**Table 2:** Split-plot central composite RSM design matrix with un-coded values of the factors and secondary structure responses.

Run	A: Pressure Level	B: Time	C: Concentration	Protein Aggregates	Beta-Sheets	Random Coil	Alpha-Helices	Beta- Turns	Antiparallel Beta- Sheets	Beta-Sheets Aggregates
	MPa	min	%	%	%	%	%	%	%	%
1	300	10	15	5.22 ± 0.22	2.73 ± 0.03	2.73 ± 0.20	16.52 ± 073	26.21 ± 2.10	39.20 ± 0.30	5.71 ± 1.54
2	300	20	25	8.47 ± 0.20	15.71 ± 0.08	9.05 ± 0.25	14.12 ± 0.79	22.09 ± 1.83	26.07 ± 0.43	4.49 ± 1.55
3	300	10	25	6.22 ± 0.21	16.22 ± 0.12	14.40 ± 0.32	14.26 ± 0.85	22.08 ± 1.86	19.96 ± 0.95	5.55 ± 0.92
4	300	20	15	6.62 ± 0.09	26.65 ± 0.25	7.47 ± 0.75	11.34 ± 0.90	23.25 ± 1.32	20.89 ± 0.99	3.78 ± 0.95
5	400	15	20	5.93 ± 0.93	18.62 ± 0.84	6.99 ± 0.94	11.63 ± 0.82	28.77 ± 0.72	23.80 ± 1.95	1.46 ± 0.09
6	400	15	20	6.00 ± 0.95	18.87 ± 0.89	7.00 ± 0.90	11.85 ± 0.80	28.68 ± 0.70	23.87 ± 1.90	1.52 ± 0.10
7	400	15	20	5.87 ± 0.90	18.94 ± 0.90	6.89 ± 0.91	11.70 ± 0.79	28.96 ± 0.74	23.97 ± 1.83	1.46 ± 0.13
8	500	20	15	7.55 ± 0.19	15.26 ± 0.84	4.94 ± 1.94	13.68 ± 0.51	27.14 ± 0.80	21.50 ± 0.93	6.95 ± 0.92
9	500	20	25	8.52 ± 0.29	16.35 ± 0.80	10.17 ± 1.38	14.04 ± 0.48	22.74 ± 0.95	20.54 ± 0.95	6.41 ± 0.32
10	500	10	25	4.62 ± 0.39	20.75 ± 0.87	3.40 ± 1.29	11.10 ± 0.72	31.28 ± 0.52	28.85 ± 0.23	0.00 ± 0.64
11	500	10	15	9.31 ± 0.91	11.43 ± 0.90	7.37 ± 1.82	16.93 ± 0.57	29.55 ± 0.92	23.03 ± 0.21	2.37 ± 0.52
12	227	15	20	8.02 ± 1.24	18.45 ± 1.45	5.62 ± 0.64	10.81 ± 1.92	24.56 ± 0.92	28.57 ± 0.29	0.79 ± 0.14
13	227	15	20	8.00 ± 1.29	18.75 ± 1.50	5.97 ± 0.61	11.02 ± 1.90	24.85 ± 0.85	28.21 ± 0.28	0.87 ± 0.19
14	400	15	20	5.93 ± 1.03	18.62 ± 0.90	6.99 ± 0.90	11.63 ± 0.89	28.77 ± 0.70	23.80 ± 1.92	1.46 ± 0.11
15	400	15	20	6.00 ± 0.92	18.53 ± 0.85	7.00 ± 0.91	11.69 ± 0.82	28.97 ± 0.77	23.98 ± 1.94	1.50 ± 0.09
16	400	15	20	6.10 ± 0.94	17.93 ± 0.91	6.90 ± 0.92	11.36 ± 0.85	28.64 ± 0.93	23.85 ± 1.91	1.48 ± 0.12
17	400	15	20	5.93 ± 0.96	18.09 ± 0.87	6.73 ± 0.90	11.63 ± 0.80	28.76 ± 0.84	23.95 ± 1.90	1.46 ± 0.09
18	400	15	20	5.87 ± 0.90	18.69 ± 0.88	7.12 ± 0.92	11.85 ± 0.89	28.32 ± 0.72	24.00 ± 1.89	1.45 ± 0.12
19	400	15	20	5.90 ± 0.92	18.42 ± 0.90	7.04 ± 0.92	11.73 ± 0.90	29.00 ±0.73	23.99 ± 1.90	1.49 ± 0.12
20	400	24	20	8.13 ± 1.43	13.47 ± 1.68	10.66 ± 1.39	14.19 ± 1.83	25.14 ± 1.29	23.42 ± 1.53	1.08 ± 0.81
21	400	15	29	6.81 ± 1.49	14.17 ±1.49	6.60 ± 1.92	12.89 ± 1.72	23.26 ± 1.82	25.46 ± 1.82	1.57 ± 0.98
22	400	06	20	8.79 ± 1.40	14.71 ± 0.74	9.39 ± 0.91	13.05 ± 1.74	19.09 ± 1.56	22.82 ± 1.73	1.26 ± 1.43
23	400	15	11	7.01 ± 1.00	14.29 ± 0.86	7.92 ± 1.47	9.51 ± 0.98	29.09 ± 0.98	22.95 ± 1.92	0.00 ± 1.92
24	573	15	20	5.41 ± 0.83	11.32 ± 1.23	8.14 ± 0.92	10.28 ± 1.02	38.78 ± 0.12	20.95 ± 0.82	0.00 ± 0.91
25	573	15	20	5.60 ± 0.82	11.45 ±1.20	8.87 ± 0.90	9.57 ± 1.00	37.94 ± 0.10	21.23 ± 0.80	0.00 ± 0.82
Control	--	--	20	14.04 ± 0.42	21.69 ± 0.28	9.93 ± 0.27	21.23 ± 0.43	7.99 ± 0.4	6.68 ± 0.32	0.74 ± 0.69

**Table 3:** Model statistics and adequacy of the models for all responses.

Response	Model	*p*-Value (Whole-Plot) *	*p*-Value (Sub-Plot) **	R2	Std. Dev.
Emulsion capacity	Reduced Cubic	0.8482	0.0146	0.49	0.1669
Emulsion stability	Reduced Cubic	0.0214	0.0145	0.84	0.4857
Protein aggregates	Reduced Cubic	0.0004	<0.0001	0.91	1.2701
Beta-sheets	Cubic	0.0116	<0.0001	1.00	4.3758
Random coil	Cubic	0.0361	<0.0001	0.99	2.3127
Alpha-helices	Reduced Cubic	0.5848	<0.0001	0.99	1.8828
Beta-turns	Quadratic	0.0020	0.0177	0.85	4.4858
Antiparallel beta-sheets	Cubic	0.0081	<0.0001	1.00	3.8812
Beta-sheets aggregates	Reduced Quadratic	0.6486	<0.0001	0.85	2.0609
Temperature of Denaturation (T_d_)	2FI	0.0069	0.0413	0.91	4.2
Enthalpy (∆H)	Linear	0.0014	0.2259	0.95	17.1

* Whole-plot: Pressure level; ** Sub-plot: Pressurization time, aquafaba concentration %, and their interaction with whole-plot.

**Table 4:** Polynomial mathematical models with interaction terms obtained in terms of coded factors for different responses.

Response	Equation
Emulsion capacity	+4.91 + 0.0074.47 × a + 0.22 × C − 0.11 × aC + 0.015 × C^2^ − 0.070 × C^3^
Emulsion stability	+1.99 + 0.99 × a − 0.17 × B − 0.14 × C + −0.076 × a^2^ + −0.068 × C^2^ − 0.42 × a^3^ + 0.13 × C^3^
Protein aggregates	+5.95 + 1.01 × a + 1.18 × B − 0.087 × C − 0.82 × aC + 0.81 × BC + 0.24 × a^2^ + 0.77 × B^2^ + 0.26 × C^2^ − 0.58 × a^3^ − 0.46 × B^3^
Beta-sheets	+18.52 − 2.08 × a − 0.36× B − 0.036 × C − 3.00 × aB + 0.98 × aC − 4.08 × BC − 1.11 × a^2^ − 1.21 × B^2^ − 1.16 × C^2^ + 2.03× aBC + 3.21 × a^2^B + 1.66 × a^2^C + 2.39 × aB^2^
Random coil	+6.96 + 0.78 × a + 0.37 × B − 0.38 × C + 0.62 × aB − 1.50 × aC − 0.11 × BC + 0.021× a^2^ + 0.87 × B^2^ − 0.058 × C^2^ + 2.41× aBC + 0.098 × a^2^B + 2.19 × a^2^C + −1.75 × aB^2^
Alpha-helices	+11.31 − 0.23 × a − 1.22 × B − 1.42 × C + 0.62 × aB − 0.75 × aC + 1.40 × BC + 1.23 × B^2^ + 0.42 × C^2^ + 0.52 × B^3^ + 0.80 × C^3^
Beta-turns	+28.76 + 3.23 × a − 0.24× B − 1.29 × C − 1.00 × aB + 0.33 × aC − 0.39 × BC + 0.81 × a^2^ − 2.44 × B^2^ − 1.09 × C^2^
Antiparallel beta-sheets	+23.91 − 2.11× a + 0.17 × B + 0.73 × C + 0.29× aB + 2.37 × aC + 2.21 × BC + 0.33 × a^2^ − 0.047 × B^2^ + 0.32 × C^2^ − 3.90 × aBC − 2.93 × a^2^B − 1.88× a^2^C + 0.58 × aB^2^
Beta-sheets aggregates	+1.90 + −0.30 × a + 0.55 × B + 1.75 × aB
Temperature of denaturation (T_d_)	+112.43 − 3.85 × a − 1.02 × B − 0.13 × C + 0.90 × aB − 0.73 × aC − 0.19 × BC
Enthalpy (∆H)	+94.18 − 16.03 × a − 1.03 × B + 1.41 × C

where a = Pressure level; B = Pressurization time; C = Aquafaba concentration.

## Data Availability

Data available on request due to restrictions.

## Supplementary Materials

The following are available online, DSC thermogram showing the enthalpy of denaturation (Td) for multiple runs (run # 12, 15, 24, and control).
