# Supplementary material for: Evaluation of Changes in Protein Quality of High-Pressure Treated Aqueous Aquafaba
Source: Molecules. 2021 Jan 5;26(1):234. doi: 10.3390/molecules26010234 (PMC7795008; doi:10.3390/molecules26010234)
Supplement: Supplementary file 1 [file molecules-26-00234-s001.pdf]

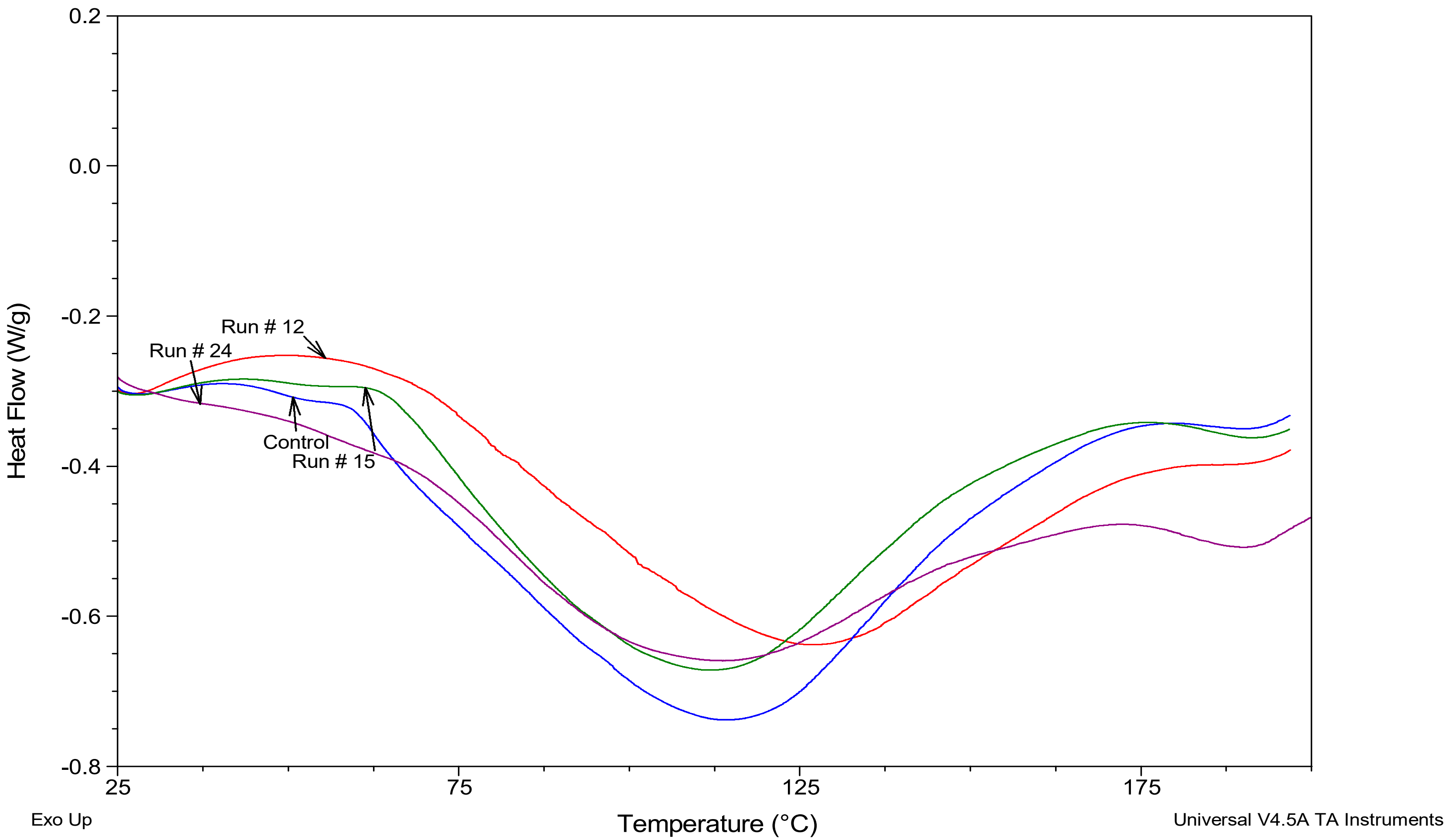

Figure. DSC thermogram showing the enthalpy of denaturation (T_d) for multiple runs (run # 12, 15, 24, and control)
